# Lack of association between Lewis phenotypes and ischemic heart disease

**DOI:** 10.1590/S1516-31802012000500012

**Published:** 2012-11-13

**Authors:** Antonio Padua Mansur, Márcia Cristina Novaretti, Solange Desiree Avakian, José Antonio Ramires

**Affiliations:** I MD, PhD. Associate Professor, Instituto do Coração (InCor), Hospital das Clínicas, Faculdade de Medicina da Universidade de São Paulo (HCFMUSP), São Paulo, Brazil.; II MD. Physician and Assistant Professor, Clinical Department, Hospital das Clínicas, Faculdade de Medicina da Universidade de São Paulo (HCFMUSP), São Paulo, Brazil.; III MD. Assistant Professor, Instituto do Coração (InCor), Hospital das Clínicas, Faculdade de Medicina da Universidade de São Paulo (HCFMUSP), São Paulo, Brazil.; IV MD, PhD. Titular Professor, Instituto do Coração (InCor), Hospital das Clínicas, Faculdade de Medicina da Universidade de São Paulo (HCFMUSP), São Paulo, Brazil.

The Copenhagen Male Study found an increased risk of ischemic heart disease in men with the Lewis blood group phenotype Le(a-b-).^1^ This phenotype has also been associated with insulin-resistance syndrome, higher body mass index and hypertriglyceridemia in men but not in women.^2^ Alcohol consumption and a high level of leisure time physical activity have been found to be protective against ischemic heart disease in Le(a-b-) men.^3^^,^^4^ Two polymorphic loci, *Se* and *Le*, contribute to the synthesis of Lewis antigens, resulting in three phenotypes: Le(a-b-), Le(a-b+) and Le(a+b-). *Se* and *Le* genes have been mapped to chromosome 19, where they are probably distant and without linkage to one another. These genes code for different fucosyltransferases that determine where fucose residues are placed on the oligosaccharide epitope. Plasma oligosaccharides are passively adsorbed to the red blood cell membrane. Because some patients may be erroneous diagnosed as having phenotype Le(a-b-) from blood assays, Lewis antigens should be checked in saliva. These antigens may be present in the saliva of secretor patients (*Se/Se*, *Se/se*) with misdiagnosed blood Le(a-b-) phenotype.^5^

We conducted a prospective, cross-sectional, age-adjusted study on 391 patients: 130 normal individuals and 261 non-diabetic patients with coronary artery disease (CAD), of whom 108 had stable CAD and no previous myocardial infarction (MI) and 153 had acute MI. Control subjects were selected from a healthy population and had a normal medical history, physical examination and resting electrocardiogram (ECG). The diagnosis of acute myocardial infarction was made if the patient fulfilled two of the following criteria: (a) typical chest pain lasting more than 20 minutes; (b) ECG on enrollment showing segment ST-T changes with or without new Q wave in 2 or more consecutive leads of serial ECGs; and, (c) elevation of serum creatine kinase-MB (CKMB) levels above 20 IU/l sampled every 6 hours within 48 hours after onset of chest pain. Clinical features, risk factors and lipid profiles were analyzed. Lewis phenotypes were evaluated in blood and saliva using the hemagglutination technique with monoclonal Lewis a and b antibodies. The Ethics Committee of Instituto do Coração (InCor), Hospital das Clínicas, Faculdade de Medicina da Universidade de São Paulo (HCFMUSP), approved the study protocol.

As shown in Table 1, the prevalence of the various Lewis phenotypes in blood and saliva was not associated with any clinical condition. Our data suggest that in this Brazilian population, blood and salivary Le(a-b-) phenotype was not associated with CAD, acute MI, or even lipid profile. Our results are consistent with the recent study by Cakir et al., who were unable to show any association between the Le(a-b-) phenotype and coronary artery disease.^6^ They also did not find any association between the Le(a-b-) phenotype and subclinical atherosclerosis. In their case-control study, they showed that the distribution of Lewis genotypes and haplotypes was the same between individuals with carotid intima-media thickness (IMT) > 1.0 mm and their controls. Nevertheless, studies have shown that the Le(a-b-) phenotype is associated both with hypertriglyceridemia and with increased serum levels of factor VIII and von Willebrand factor. Cakir et al.^6^ stated that these associations could favor future atherothrombotic disease. Future studies will be needed in order to establish what influence Lewis phenotypes have on Brazilian populations.

**Table 1.** Prevalence of Lewis phenotypes among normal Brazilians, those with stable coronary artery disease (CAD), and those with acute myocardial infarction (MI)

	Normal (n = 130)	Stable CAD (n = 108)	Acute MI (n = 153)
Serum assay			
Le(a-b-)	32 (24.6%)	19 (17.6%)	43 (28.1%)
Le(a-b+)	71 (54.6%)	71 (65.7%)	78 (51.0%)
Le(a+b-)	27 (20.8%)	18 (16.7%)	32 (20.9%)
Saliva assay			
Non-secretor	32 (24.6%)	19 (17.6%)	39 (25.5%)
Lea	27 (20.8%)	18 (16.7%)	30 (19.6%)
Leb	48 (36.9%)	47 (43.5%)	58 (37.9%)
Lea, Leb	23 (17.7%)	24 (22.2%)	26 (17.0%)

This study was supported by funds from the State of São Paulo Department of Science, Technology and Economic Development (Secretaria de Ciência e Tecnologia e Desenvolvimento Econômico do Estado de São Paulo).
